# Warm‐Water Regimes Influence Microbial Diversity and Ecological Functions in Subtropical Gulf

**DOI:** 10.1002/ece3.73435

**Published:** 2026-04-09

**Authors:** Qing He, Laizhen Huang, Chongqiu Huang, Rajapakshalage Thashikala Nethmini, Jannatul Ferdoush, Shengyao Zhou, Gonglingxia Jiang, Qinghua Hou, Xiaolei Li, Qingxiang Chen, Ke Dong, Lingling Xie, Nan Li

**Affiliations:** ^1^ Key Laboratory of Climate, Resources and Environment in Continental Shelf Sea and Deep Sea of Department of Education of Guangdong Province, Department of Oceanography, Key Laboratory for Coastal Ocean Variation and Disaster Prediction, College of Ocean and Meteorology Guangdong Ocean University Zhanjiang China; ^2^ College of Environmental Science and Engineering Guilin University of Technology Guilin China; ^3^ Department of Biological Sciences Kyonggi University Suwon‐si Gyeonggi‐do South Korea

**Keywords:** ecological function, indicator species, marine ecosystem, microbial diversity, warm‐water dynamics

## Abstract

The marine ecosystem is a key biogeochemical engine driven by complex hydrodynamic processes and harbors significant microbial abundance and diversity. However, the influence of warm‐water dynamics on marine microbial community composition and ecological function remains unclear. In this study, we used high‐throughput sequencing to investigate the impact of water temperature on the composition and ecological processes of marine microbial communities in the Beibu Gulf. We found that temperature variation strongly influenced microbial community composition and ecological function in seawater. Proteobacteria were enriched in warm zones, whereas Cyanobacteria abundance decreased as the temperature increased. The transition zone exhibited the highest alpha diversity in summer and winter, suggesting favorable ecological niches under intermediate conditions. Functional analysis indicated that metabolic pathways dominated, particularly in the transition and warm zones in summer and winter. Random Forest analysis revealed *Synechococcus* sp. RCC307 had the highest relative abundance in the warm water zone in spring. *Fulvimarina pelagi* HTCC2506 was a summer warm‐water specialist, and *Acinetobacter* sp. PmeaMuc16 and *Agrobacterium radiobacter* were warm‐water indicator species in winter. Overall, these findings emphasize the critical role of thermal regimes in shaping bacterial communities in subtropical marine ecosystems and provide new insights into predicting microbial responses under ocean warming scenarios.

## Graphical abstract

In this study, high‐throughput sequencing was employed to investigate the impact of water temperature on the composition and ecological functions of marine microbial communities in the Beibu Gulf. It was found that temperature variations significantly influenced the composition and ecological functions of these microbial communities. Specifically, different temperature zones harbored distinct dominant microbial species, highlighting the critical role of thermal regimes in shaping bacterial communities within subtropical marine ecosystems.

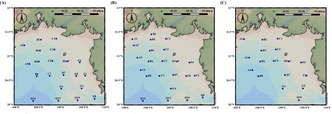

## 1 Introduction

The marine environment is the most extensive ecosystem on Earth that plays a pivotal role in global biochemical cycles and sustains the greatest microbial abundance and diversity of any natural system (Guo et al. 2016). As the most sensitive indicators of environmental change, microbial communities are directly exposed to environmental fluctuations (Labbate et al. 2016; Zhang et al. 2016). Recent studies have indicated that environmental changes significantly influence the marine bacterial communities, driving microbial turnover and species diversity across diverse maritime regions. For example, Li et al. (2021) found that the community structure of marine bacteria responds to the Chlorophyll‐*a* concentrations and N/P ratios in the Eastern Indian Ocean. Ren et al. (2019) found that the composition of marine planktonic bacterial communities across different temperatures exhibited high heterogeneity in the Daya Gulf. Therefore, elucidating how environmental variability shapes microbial communities and functions is key to predicting marine ecosystem resilience and biogeochemical feedback under global climate change.

Warm water in the ocean typically exhibits high temperatures and low salinity compared to surrounding waters. The warmer water is usually located on the surface of the ocean and is significantly affected by seasonal changes and ocean currents (Moon et al. 2019; Sanchez‐Cabeza et al. 2022). The formation of warm water is influenced by climate change and certain closely related factors such as ocean circulation, wind speed, and vertical mixing of seawater (Reader 2022). In subtropical and tropical regions, strong solar radiation, high evaporation rates, and warm climatic conditions all contribute to the formation of warm water (Allan et al. 2020). Multiple studies have shown that water temperature has a significant impact on microbial communities in different habitats. Zhou et al. (2022) found that *Thermococcus* and other thermophilic archaea dominate in deep‐sea hydrothermal vent environments, whereas other microbial groups prevail in low‐temperature aquatic habitats. Ilicic et al. (2023) reported that a 1°C increase in Antarctic environments can rapidly reshape bacterial communities, highlighting their extreme thermal sensitivity. However, the impact of water temperature variation on the composition and ecological processes of marine microbial communities in subtropical surface seawater remains unclear.

Environmental changes strongly shape microbial communities, which actively adjust via changes in composition and function (Manzoor et al. 2021). Microbial communities are often used as sensitive bioindicators of environmental change in marine ecosystems. For example, Li et al. (2020) found that *Ponticaulis koreensis* , *Nautella italic*, *Anaerospora hongkongensis*, *Candidatus Aquiluna rubra*, *Roseovarius pacificus* , and *Vibrio shilonii* can be used as bioindicators for trophic levels in the Beibu Gulf. Chen et al. (2022) found that small chromophytic phytoplankton (SCP) communities could be considered as bioindicators for evaluating eutrophic states in estuarine and coastal ecosystems. Warm‐adapted species generally grow and reproduce at higher temperatures in subtropical water (Poudel et al. 2014). Therefore, we hypothesized that the microbial communities differed among the warm, transition, and non‐warm zones in seawater.

The Beibu Gulf, a subtropical gulf located in the northwest region of the South China Sea, has abundant marine resources and complex microbial community structures (Ma et al. 2010). Therefore, to test our hypothesis, we collected seawater samples in the Beibu Gulf to (i) reveal the effect of warm water on the structure of marine bacterial communities; (ii) explore the impact of warm water on marine bacterial ecological function; and (iii) identify and characterize the bacterial species adapted to warm‐water conditions. Collectively, this study provides a comprehensive understanding of how temperature variation shapes both the taxonomic composition and functional potential of marine bacterial communities in the surface waters of the Beibu Gulf, and offers critical insights into the mechanisms maintaining marine ecosystem stability.

## 2 Materials and Methods

### 2.1 Sampling Sites and Environmental Parameters

**FIGURE 1:**
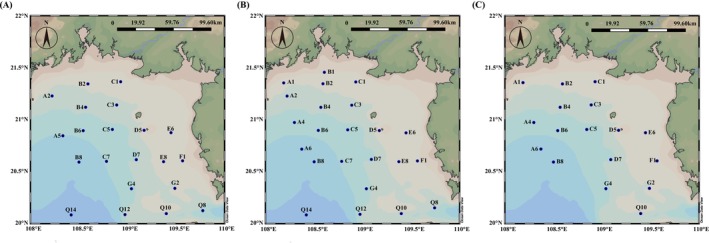
(A–C) Location of sampling sites in the Beibu Gulf area in spring, summer, and winter, respectively.

**FIGURE 2:**
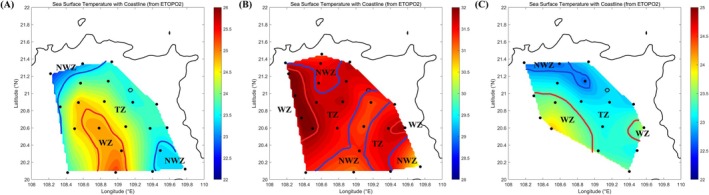
(A–C) Location and water temperature distribution of sampling sites in the Beibu Gulf area in spring, summer, and winter, respectively. NWZ, non‐warm zone; TZ, transition zone; WZ, warm zone.

A total of 61 sites were used in this study. Samples were collected from seawater at a depth of 3 m during open cruises in the Beibu Gulf in April, August, and December 2023 (Figure 1). Seawater samples collected were divided seasonally into three categories: 21, 23, and 17 sites in spring, summer, and winter, respectively. In each season, samples were divided into the warm, transition, and non‐warm zones according to the water temperature during collection (Figure 2). Three replicate water samples were collected using a SBE 32 Carousel Water Sampler at each site. All samples were stored at 4°C before bacteria isolation and analysis of environmental factors. For the bacterial community analysis, a vacuum pump was employed to sequentially filter 1 L of seawater from each sample through 3‐μm sieves (Port Washington, NY, USA) to eliminate debris and larger organisms. The filtered samples were subsequently collected on 0.22‐μm polycarbonate membranes (Millipore Corporation, Billerica, MA, USA). Environmental parameters of the samples were assessed using a portable meter (556 MPS; YSI, USA) to measure temperature, salinity, pH, and dissolved oxygen (DO). In addition, the concentrations of phosphate (${\mathrm{PO}}_{4}^{3-}$‐P), nitrite (${\mathrm{NO}}_{2}^{-}$‐N), nitrate (${\mathrm{NO}}_{3}^{-}$‐N), and ammonium (${\mathrm{NH}}_{4}^{+}$‐N) were determined using a continuous flow analyzer (Seal‐AA3, Germany). Chlorophyll‐*a* (Chl‐*a*) content was measured using a previously described method (Association 1926). Total organic carbon (TOC) was quantified using a TOC‐VCPH analyzer (Shimadzu, Japan), and chemical oxygen demand (COD) was measured using alkaline potassium permanganate (KMnO₄). Dissolved inorganic nitrogen (DIN) was calculated as the sum of ${\mathrm{NO}}_{2}^{-}$‐N, ${\mathrm{NO}}_{3}^{-}$‐N, and ${\mathrm{NH}}_{4}^{+}$‐N, whereas dissolved inorganic phosphorus (DIP) was represented by ${\mathrm{PO}}_{4}^{3-}$‐P.

### 2.2 DNA Extraction and PCR Amplification

Genomic DNA extraction from seawater samples was performed using the DNeasy PowerWater Kit (QIAGEN, USA) in conjunction with 0.22‐μm polycarbonate membranes, following the manufacturer's instructions. The V3–V4 regions of the 16S rRNA genes were amplified using the primer set 16S‐F (5′‐AGAGTTTGATCMTGGCTCAG‐3′) and 16S‐R (5′‐TACGGYTACCTTGTTACGACTT‐3′) (Cole et al. 2013). The PCR mixture (20 μL) comprised 2 μL of DNA template, 6 μL of ddH_2_O, 10 μL of 2 × Taq PCR Mastermix (TianGen, China), and 2 μL each of the forward and reverse primers. PCR amplification was performed using a Bio‐Rad thermocycler (Hercules, CA, USA) as follows: an initial activation step at 94°C for 1 min, followed by 35 cycles of denaturation at 95°C for 30 s, annealing at 56°C for 30 s, and extension at 72°C for 30 s, with a final elongation step at 72°C for 10 min. Ultrapure water served as the negative control to eliminate the potential for false‐positive results. The PCR products were validated using 2% agarose gel electrophoresis and visualized under ultraviolet (UV) light with a gel imaging system.

### 2.3 High‐Throughput Sequencing

A clean library was generated following the standard Illumina library preparation protocols and subsequently sequenced on the Illumina MiSeq platform (Majorbio Co. Ltd., Shanghai, China). Sequences containing low‐quality reads were filtered using the DADA2 denoising algorithm within the Qiime2 framework (Caporaso et al. 2010). For downstream analyses, amplicon sequence variants (ASVs) derived from the Illumina amplicon dataset were employed without applying arbitrary dissimilarity thresholds (Callahan et al. 2017). Taxonomic assignment of ASVs was conducted by performing a local BLASTN search (with a cutoff E‐value of 1e^−10^) against the Silva 16S database (Sun et al. 2022). All sequence data are deposited in GenBank under the BioProject Accession number PRJNA1335633.

### 2.4 Statistical Analysis

Statistical analyses were performed using the R 4.3.2 software. A water temperature distribution map was constructed in MATLAB 2024a. The significant difference among the communities was determined by permutational multivariate analysis of variance (PERMANOVA). To visualize the taxonomic composition of the bacterial community, a ternary diagram was constructed in R using the “Ternary” package to illustrate the relative abundances of the 10 most prevalent bacterial phyla. We calculated the Richness, Shannon, Simpson, and Chao1 using the “vegan” package and visualized using the “ggplot2” package, and the Alpha diversity was represented using the Shannon index (Good 1953; Kemp and Aller 2004). Nonmetric multidimensional scaling (NMDS) and analysis of similarity (ANOSIM) were used to estimate the difference in community structure between different samples based on the Bray–Curtis dissimilarity using the “vegan” package and visualized using the “ggplot2” package. Network analyses of prokaryotes were performed using the “Hmisc” package and visualized using Gephi 0.9.2 software. Bacterial community functional analysis was conducted using PICRUSt2 and visualized using the “pheatmap” package in R. The twenty most important indicator species for classification were identified through a random “RandomForest” package.

## 3 Results

### 3.1 Delineation of the Warm‐Water Zone and Geochemical Analysis

In this study, 183 water samples from different seasons were collected from the Beibu Gulf. Based on the sample temperatures during collection, we categorized them seasonally into three zones: warm, transition, and non‐warm zones, with each temperature interval set at 0.5°C (Figure 2). The PERMANOVA (*r* = 0.300, *p* = 0.001) test indicated a significant difference in the temperature zones across the three seasons. The physicochemical parameters of environmental factors in the Beibu Gulf during spring, summer, and winter are presented in Table S1. In winter, the average seawater salinity, dissolved oxygen (DO) concentration, and pH were higher than those in spring and summer. In spring and winter, the salinity exhibited an upward trend as the temperature increased. In spring, the concentrations of ${\mathrm{NO}}_{2}^{-}$‐N, ${\mathrm{NO}}_{3}^{-}$‐N, and Chl‐*a* first increased and then decreased with rising temperatures, and the Chl‐*a* concentration in winter followed the same pattern.

### 3.2 Composition and Diversity of Marine Bacteria in the Beibu Gulf

**FIGURE 3:**
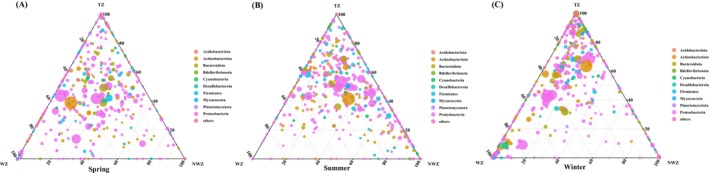
(A‐C) Ternary plots depicting the relative abundance of marine bacteria at the phylum level across three temperature zones (warm, transition, and non‐warm zones). NWZ, non‐warm zone; TZ, transition zone; WZ, warm zone.

We obtained 1,456,439 high‐quality sequences. At the phylum level, Proteobacteria (49.11%) were dominant, followed by Cyanobacteria (29.97%) (Figure 3). In spring, the relative abundance of both Proteobacteria and Cyanobacteria increased as the temperature increased. In summer, Proteobacteria (50.20%) and Cyanobacteria (29.18%) had the highest and lowest relative abundances in the transition zone, respectively. In winter, the relative abundance of Cyanobacteria decreased as the temperature increased, and Proteobacteria (65.22%) had the highest relative abundance in the warm zone.

**FIGURE 4:**
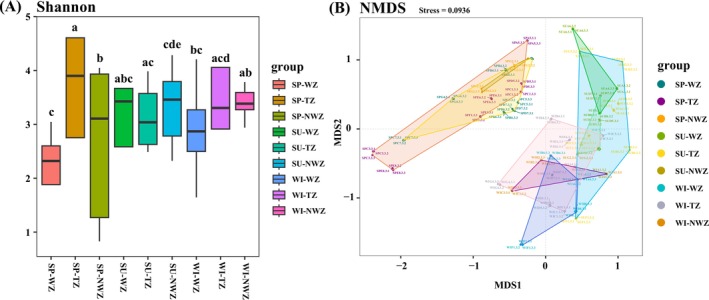
(A) Alpha diversity (Shannon index) presented in boxplots; (B) Beta dissimilarity (Bray–Curtis distance) presented by a non‐metric multidimensional scaling (NMDS) plot. NWZ, non‐warm zone; SP, spring; SU, summer; TZ, transition zone; WI, winter; WZ, warm zone.

The Shannon index was used to assess the alpha diversity of marine bacteria. The results showed that alpha diversity increased with the temperature decrease in spring, whereas the transition zone had the highest alpha diversity in summer and winter (Figure 4A). A non‐metric multidimensional scaling (NMDS) plot based on the Bray–Curtis distance revealed the variation between the marine bacteria communities from all habitats (Figure 4B). These results indicated that there are significant differences in the diversity of bacterial communities across different temperature zones in different seasons.

### 3.3 Ecological Function of Marine Bacteria in the Beibu Gulf Under Different Thermal Regimes

**FIGURE 5:**
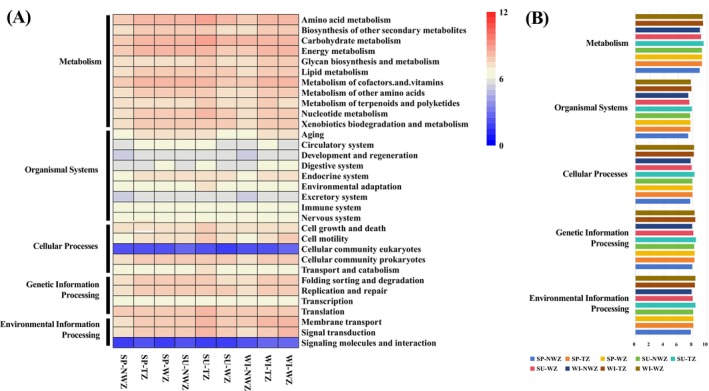
(A) Ecological functional profiles of bacterial communities in the Beibu Gulf based on the PICRUSt2 analysis, selecting categories of level 2 KEGG pathways. (B) Relative abundance of the functional profiles of bacterial communities in Beibu Gulf. KEGG, Kyoto Encyclopaedia of Genes and Genomes; NWZ, non‐warm zone; SP, spring; SU, summer; TZ, transition zone; WI, winter; WZ, warm zone.

To understand the response of bacterial communities to differences in temperature and the shifts in community structure, we created predictive functional profiles of bacterial communities in the Beibu Gulf based on the PICRUSt2 analysis. The results showed that the relative abundance of metabolism (81.31%) was much higher than the other functional profiles (Figure 5A). In addition, the relative abundance of metabolism, organismal systems, cellular processes, genetic information processing, and environmental information processing pathways is higher in the warm water and transition zones in spring and winter, whereas the transition zone in summer had the highest relative abundance for the abovementioned functional pathways. The results showed that pathways such as carbohydrate, amino acid, and energy metabolism showed higher abundance (Figure 5B). The heatmap of level 2 KEGG pathways also showed that pathway abundance was higher in the warm zone in spring, whereas the transition zone had the highest pathway abundance in summer and winter (Figure 5B). These findings indicate that perturbations induced by variations in water temperature could result in shifts in the bacterial community composition and the functional gene profiles of bacteria within the subtropical Beibu Gulf.

### 3.4 Warm‐Adapted Species of Marine Bacteria in the Beibu Gulf

**FIGURE 6:**
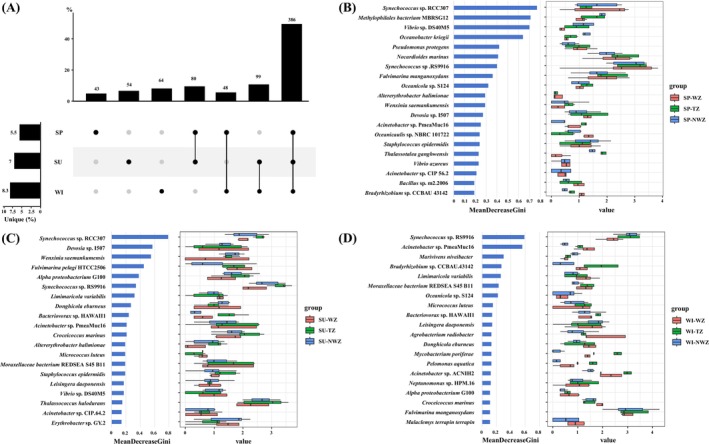
(A) The UpSet plot shows the common and unique species across the different seasons. (B–D) Random Forest classification of the top 30 important species in spring, summer, and winter, respectively. Left: The top 20 taxa analyzed using the Gini index, representing the importance of each species in different groups. Right: Relative abundances of the top 20 species. SP, spring; SU, summer; TZ, transition zone; NWZ, non‐warm zone; WI, winter; WZ, warm zone.

A total of 774 species were found in the subtropical Beibu Gulf. The UpSet plot showed that the collective species among the three seasons account for the highest proportion. In addition, specific species account for 5.56%, 6.98%, and 8.27% in spring, summer, and winter, respectively. The Random Forest analysis was performed to identify the bioindicators in warm water zones across the three seasons (Figure 6). The 20 most important indicator species were displayed based on their maximal Gini values in each season. *Synechococcus* sp. RCC307 exhibited the highest Gini values in both spring and summer and was consistently the pivotal indicator species in spring warm water. In summer, *Synechococcus* sp. RCC307, *Devosia* sp. I507, *Wenxinia saemankumensis*, and *Fulvimarina pelagi* HTCC2506 had high Gini values, and their abundances were different in the warm‐water, transition, and non‐warm water zones. However, only *F. pelagi* HTCC2506 had higher abundance in the warm‐water zone than the transition and non‐warm water zones. *Acinetobacter* sp. PmeaMuc16 and *Agrobacterium radiobacter* could be warm‐water indicator species in winter. *Synechococcus* sp. RS9916 had high Gini values and abundances in all three seasons, but the abundance in the warm‐water zone was lower than that in the transition and non‐warm water zones in each season. These results indicated that there are different warm‐adapted species in the different seasons.

## 4 Discussion

### 4.1 Water Temperature Regulates the Physicochemical Properties and Microbial Community in Seawater

Temperature is one of the most important environmental parameters in marine ecosystems. It not only directly influences the physicochemical properties of seawater, but also indirectly shapes the habitat and reproduction of marine microorganisms (Tonelli et al. 2021). In marine systems, temperature variation can regulate nutrient cycling and primary productivity, thereby driving the composition, function, and interaction networks of bacterial communities (Carozza et al. 2019; Dai et al. 2022). In particular, the occurrence of warm‐water zones, often formed through seasonal change or regional hydrodynamics, represents a distinct thermal niche that strongly modulates microbial community. Warm‐water zones can selectively favor thermotolerant or warm‐adapted taxa, alter metabolic pathways, and influence biogeochemical cycling; thus, understanding microbial responses to climate‐driven ocean warming is crucial (Walker et al. 2024). Therefore, investigating the correlated variation of temperature and seawater physicochemical properties is essential for understanding microbial ecological processes.

In the present study, the average salinity, DO, and pH in winter were higher than those in spring and summer. In both spring and winter, salinity increased with increasing temperature. In spring, ${\mathrm{NO}}_{2}^{-}$‐N, ${\mathrm{NO}}_{3}^{-}$‐N, and Chl‐*a* concentrations first increased and then decreased with temperature, and a similar pattern was observed for Chl‐*a* in winter. These patterns indicate that temperature variation, which influences water column stratification and mixing, alters the vertical distribution of nutrients and bacterial productivity, thereby indirectly affecting bacterial community structure and function (Cavicchioli et al. 2019). For example, moderate warming can stimulate short‐term bacterial blooms, increasing organic matter input and providing abundant carbon sources for bacterial communities. However, excessive warming or nutrient depletion may reduce bacterial productivity, leading to shifts in bacterial composition and metabolic functions (Engel et al. 2011). In addition, elevated seawater temperatures can intensify vertical stratification by increasing the density difference between surface and deeper layers. This enhanced stratification reduces vertical mixing, thereby restricting the upward transport of nutrient‐rich deep water to the surface. Consequently, nutrient availability in surface waters declines, potentially constraining primary production, subsequently limiting the organic matter supply that supports heterotrophic bacterial growth (Engel et al. 2011; Sunagawa et al. 2015). Thus, temperature plays a key regulatory role in the physicochemical properties and microbial communities of seawater in the Beibu Gulf ecosystem.

### 4.2 Temperature Variation Influences Bacterial Structure and Diversity in the Beibu Gulf

Proteobacteria and Cyanobacteria were the predominant phyla in seawater samples from the Beibu Gulf area. Recently, several studies have been conducted on marine bacteria, particularly in subtropical gulfs affected by anthropogenic activities (Yang et al. 2024). For example, Peng et al. (2024) found that Proteobacteria was the dominant bacterial phylum in the Beibu Gulf in both the wet and dry seasons, followed by Bacteroidetes and Actinobacteria. Li et al. (2020) reported that Alphaproteobacteria and Gammaproteobacteria are the most abundant bacterial species in the Beibu Gulf seawater samples across a eutrophication gradient. Our results indicated that Proteobacteria had the highest relative abundance in the transition zone, whereas Cyanobacteria had the lowest relative abundance in this zone in summer. In winter, the relative abundance of Cyanobacteria decreased with increasing temperature, whereas that of Proteobacteria peaked in the warm‐water zone. This shift in community structure suggests these bacteria have distinct ecological adaptation strategies to temperature changes (Paerl and Paul 2012). Proteobacteria dominated throughout the year and exhibited a marked increase in relative abundance in warmer waters, particularly in the warm‐water zone during winter. This pattern may be attributed to their high metabolic versatility and efficient utilization of organic matter, enabling rapid proliferation under elevated temperatures and enriched organic carbon conditions (Pinhassi and Berman 2003). In contrast, Cyanobacteria showed an increasing trend in relative abundance with increasing temperature during spring, suggesting strong adaptability to warmer environments. However, in winter, the relative abundance of Cyanobacteria declined with increasing temperature, likely due to reduced competitive advantage and decreased photosynthetic efficiency under colder, low‐light conditions (Paerl and Huisman 2008). Such differences indicate that the ecological niches of different bacterial taxa undergo dynamic shifts across temperature variations, shaping the overall community diversity patterns.

Diversity analysis further revealed the influence of temperature on community structure. In spring, the Shannon diversity index values increased as temperature decreased, and in summer and winter, the transition zone exhibited the highest diversity. This suggests that intermediate temperature conditions and their associated physicochemical properties may provide favorable ecological niches for a greater variety of bacterial species, thereby enhancing community diversity (Fuhrman et al. 2015). NMDS analysis indicated significant differences in bacterial community composition between temperature zones and seasons. These differences may be attributed not only to the direct influence of temperature on bacterial metabolism and growth rates but also to its indirect regulation of nutrient availability, light conditions, and interspecies competition, which collectively shape the community structure and diversity patterns (Cavicchioli et al. 2019). Overall, these findings underscore the pivotal role of temperature variations interacting with seasonal dynamics in shaping the structure and diversity patterns of marine bacterial communities.

### 4.3 Warm Water Shapes the Ecological Function of Marine Bacteria in Subtropical Thermal Regime Zones

Functional predictions of bacterial communities in the Beibu Gulf based on PICRUSt2 revealed that metabolism‐related pathways dominated the functional repertoire (81.31%), substantially exceeding all other functional categories. This indicates that metabolic activity plays a pivotal role in sustaining microbial ecological functions in subtropical marine ecosystems. Thermal variation was pronounced, and the relative abundance of metabolic pathways was consistently higher in summer than in spring and winter, suggesting that elevated summer temperatures promote stronger metabolic reliance to cope with accelerated biogeochemical cycling (Fuhrman et al. 2015; Sunagawa et al. 2015).

Within the Level 2 KEGG functional hierarchy, pathways such as carbohydrate, amino acid, and energy metabolism showed high relative abundance, highlighting their ecological significance in microbial adaptation (Figure 5A). For example, carbohydrate metabolism reflects the central role of bacteria in degrading phytoplankton‐derived dissolved organic carbon, especially during seasonal blooms when labile polysaccharides become abundant (Teeling et al. 2012). Falkowski et al. (2008) found that energy metabolism, particularly pathways related to oxidative phosphorylation and carbon fixation, strengthens microbial contributions to primary and secondary production. The metabolism of cofactors and vitamins is essential for microbial symbioses. For example, vitamin B12 synthesis by bacteria supports the growth of eukaryotic phytoplankton, forming tight metabolic interdependencies (Croft et al. 2005). Croft et al. (2005) found enriched translation pathways, which suggests high bacterial growth potential, consistent with enhanced microbial productivity in warmer waters. Moreover, other studies revealed that functional abundance peaked in the warm‐water zone during spring, whereas the transition zone exhibited the highest values in both summer and winter, likely reflecting differences in water column stratification, nutrient availability, and shifts in microbial composition (Galand et al. 2018; Kent 2017).

Differences across water zones in the functional composition of bacterial communities in the Beibu Gulf reflect the strong influence of temperature, water column stratification, and nutrient dynamics on microbial ecology. During summer, enhanced stratification and higher solar radiation stimulate bacteria production, increasing the release of organic exudates that promote bacterial carbohydrate metabolism (Kieft et al. 2021). Simultaneously, nitrogen limitation in surface waters favors nitrogen‐fixing groups such as Cyanobacteria, thereby increasing the contribution of nitrogen cycling processes to community function (Zehr and Kudela 2011). Elevated temperatures have also been associated with enrichment of signal transduction and stress response pathways, indicating bacterial adaptation through gene regulation and intercellular communication (Moran et al. 2017). In contrast, winter mixing replenishes nutrients and supports a shift toward amino acid and energy metabolism, often dominated by Proteobacteria adapted to cooler waters (Fuhrman et al. 2015). Taken together, these patterns demonstrate that temperature‐driven shifts in stratification, nutrient cycling, and community composition collectively shape the metabolic potential of bacterial assemblages in the Beibu Gulf.

### 4.4 Warm‐Adapted Species Reflect the Warm‐Water Zone in the Beibu Gulf

Our findings demonstrated that indicator bacterial species associated with warm water in the Beibu Gulf varied significantly among different seasons, highlighting the strong influence of temperature on community assembly. For example, *Synechococcus* sp. RCC307 emerged as the pivotal warm water indicator in both spring and summer, suggesting its ecological adaptation to elevated temperature and light conditions, which is consistent with previous studies reporting that *Synechococcus* is widely distributed in subtropical and tropical oceans and thrives in stratified, nutrient‐fluctuating waters (Flombaum et al. 2013; Partensky et al. 1999). In contrast, species such as *F. pelagi* HTCC2506 showed a higher abundance specifically in the warm water zone during summer, indicating niche specialization under seasonal thermal gradients (Cho and Giovannoni 2003). In winter, the dominance of *Acinetobacter* sp. PmeaMuc16 and *A. radiobacter* as indicator species implied that some heterotrophic bacteria may be more competitive under lower‐temperature, nutrient‐enriched conditions, which aligns with findings that temperature shifts often restructure bacterial trophic strategies and metabolic potential (Bunse and Pinhassi 2017; Cavicchioli et al. 2019).

Moreover, the consistent detection of *Synechococcus* sp. RS9916 across all three seasons, with reduced abundance in the warm water zone compared to the transition and non‐warm zones, suggested that certain species may possess broad thermal tolerances but still experience competitive exclusion in thermally stratified environments (Buitenhuis et al. 2012; Johnson et al. 2006). These differences highlight that bacterial community assembly in subtropical gulfs is governed by both environmental filtering and biotic interactions, leading to temporally dynamic shifts in species composition (Fuhrman et al. 2015; Sunagawa et al. 2015). Taken together, our findings indicate that warm water exerts a strong selective pressure on bacterial communities in the Beibu Gulf, not only shaping indicator species across seasons but also reflecting the broader principle that microbial biogeography is tightly coupled to temperature and oceanographic regimes. By transcending purely descriptive taxonomic characterization, this study identifies robust, seasonal warm‐water bioindicators. Collectively, these findings provide high‐resolution biological predictors and mechanistic functional insights, which are critical for forecasting the trajectories of marine ecosystem reorganization under projected ocean warming scenarios.

## 5 Conclusions

The present study demonstrates that water temperature variation is a key driver shaping the physicochemical properties of seawater, and the structure, diversity, and functional profiles of bacterial communities in the subtropical Beibu Gulf. Proteobacteria and Cyanobacteria dominated the bacterial assemblages, with distinct thermal adaptations. Proteobacteria dominated in warm‐water zones, whereas Cyanobacteria exhibited variable abundance patterns across seasons. Functional predictions revealed that metabolic pathways dominated, with temperature shifts reflecting changes in nutrient availability, water column stratification, and bacterial productivity. Moreover, warm‐water indicator species showed heterogeneity across seasons, with *Synechococcus* sp. RCC307 exhibiting the highest relative abundance in the warm‐water zone in spring; that of *F. pelagi* HTCC2506 was highest in summer in the warm‐water zone; and that of *Acinetobacter* sp. PmeaMuc16 and *A. radiobacter* was highest in the warm‐water zone in winter. These results highlight that temperature fluctuations directly influence microbial metabolism and growth, and also indirectly regulate community assembly through environmental filtering and biotic interactions. Overall, warm water formation exerts a selective pressure that structures bacterial communities, providing insights into microbial adaptation strategies, with valuable implications for monitoring and managing subtropical marine ecosystems under climate change.

## Author Contributions

**Qing He:** conceptualization (equal), methodology (equal), supervision (equal), writing – review and editing (equal). **Laizhen Huang:** conceptualization (equal), methodology (equal), supervision (equal), writing – review and editing (equal). **Chongqiu Huang:** writing – review and editing (equal). **Rajapakshalage Thashikala Nethmini:** writing – review and editing (equal). **Jannatul Ferdoush:** writing – review and editing (equal). **Shengyao Zhou:** writing – review and editing (equal). **Gonglingxia Jiang:** writing – review and editing (equal). **Qinghua Hou:** writing – review and editing (equal). **Xiaolei Li:** writing – review and editing (equal). **Qingxiang Chen:** writing – review and editing (equal). **Ke Dong:** conceptualization (equal), supervision (equal), writing – review and editing (equal). **Lingling Xie:** writing – review and editing (equal). **Nan Li:** conceptualization (equal), data curation (equal), formal analysis (equal), investigation (equal), methodology (equal), visualization (equal), writing – original draft (equal), writing – review and editing (equal).

## Funding

This study was jointly funded by the Guangdong Ocean University Innovative Team (Early‐warning of marine disasters) (Grant number 2023KCXTD015); National Natural Science Foundation of China's 2023 Beibu Gulf Shared Voyage; the Scientific Research Start Funds of Guang dong Ocean University; the National Research Foundation of Korea (NRF) grant funded by the Korea government (MSIT) (RS‐2022‐NR073058); Database of graphic and video images of planktonic animals in the western Guangdong waters (Grant number S202310566019); Innovation Team Project of Universities in Guangdong Province: 2025KCXTD020.

## Conflicts of Interest

The authors declare no conflicts of interest.

## Supporting information


**Table S1:** ece373435‐sup‐0001‐TableS1.docx.

**Data S1:** ece373435‐sup‐0002‐SupplementaryInformationCode.docx.

## Data Availability

All sequencing data for these studies were generated in GenBank under the number PRJNA1335633.
