# Supplementary material for: Warm‐Water Regimes Influence Microbial Diversity and Ecological Functions in Subtropical Gulf
Source: Ecol Evol. 2026 Apr 9;16(4):e73435. doi: 10.1002/ece3.73435 (PMC13063387; doi:10.1002/ece3.73435)
Supplement: Supplementary file 1 — Table S1: ece373435‐sup‐0001‐TableS1.docx. [file ECE3-16-e73435-s001.docx]

**Supplementary Information (Table S1)**

**Warm-Water Regimes Influence Microbial Diversity and Ecological Functions in Subtropical Gulf**

**Qing He^1,a^, Laizhen Huang^1,a^, Chongqiu Huang^1^, Rajapakshalage Thashikala Nethmini^1^, Jannatul Ferdoush^1^, Shengyao Zhou^2^, Gonglingxia Jiang^1^, Qinghua Hou^1^, Xiaolei Li^1^, Qingxiang Chen^1^, Ke Dong^3^, Lingling Xie^1^, Nan Li^1*^**

^1^Key Laboratory of Climate, Resources and Environment in Continental Shelf Sea and Deep Sea of Department of Education of Guangdong Province, Department of Oceanography, Key Laboratory for Coastal Ocean Variation and Disaster Prediction, College of Ocean and Meteorology, Guangdong Ocean University, Zhanjiang 524088, China

^2^College of Environmental Science and Engineering, Guilin University of Technology, Guilin, China

^3^Department of Biological Sciences, Kyonggi University, 154-42, Gwanggyosan-ro, Yeongtong-gu, Suwon-si, Gyeonggi-do 16227, South Korea; Republic of Korea

**Correspondence:**

*Corresponding Author: Nan Li

nli0417@163.com

^a^Qing He and Laizhen Huang contributed equally to this paper and are co-first authors.

**Table S1** The physicochemical parameters of environmental factors in the Beibu Gulf during spring, summer, and winter.

| sites | Temperature | Salinity | DO | NH_4_^+^ | pH | Chl-*a* | NO_2_^-^ | P^-^ | NO_3_^-^ |
| --- | --- | --- | --- | --- | --- | --- | --- | --- | --- |
| SPA2.3 | 22.66 | 32.72 | 7.89 | - | 8.13 | 0.56 | 0.03 | 7.49 | 0.65 |
| SPB2.3 | 23.16 | 32.42 | 7.41 | - | 8.14 | 1.30 | 0.70 | 9.14 | 0.56 |
| SPG2.3 | 23.38 | 32.77 | 6.67 | - | 8.10 | 0.76 | 2.53 | 7.47 | 4.34 |
| SPD5.3 | 23.61 | 32.76 | 6.57 | - | 8.06 | 0.59 | 2.72 | 9.06 | 2.55 |
| SPC1.3 | 23.64 | 32.66 | 7.21 | 0.51 | 8.06 | 2.28 | 0.80 | 11.83 | 0.95 |
| SPF1.3 | 23.72 | 32.80 | 6.81 | - | 8.10 | 1.40 | 1.41 | 9.01 | 5.18 |
| SPE8.3 | 23.73 | 32.73 | 6.74 | - | 8.09 | 1.26 | 2.29 | 8.22 | 3.87 |
| SPE6.3 | 23.85 | 32.87 | 6.67 | - | 8.09 | 1.01 | 2.30 | 10.02 | 4.85 |
| SPC3.3 | 23.89 | 32.79 | 7.56 | - | 8.14 | 2.80 | 0.27 | 8.62 | 0.80 |
| SPA5.3 | 23.94 | 33.28 | 7.17 | - | 8.16 | 0.28 | - | 5.28 | 0.51 |
| SPB4.3 | 24.02 | 33.16 | 7.95 | - | 8.18 | 0.67 | 0.01 | 2.06 | 0.56 |
| SPD7.3 | 24.45 | 33.29 | 7.06 | - | 8.12 | 1.18 | 0.27 | 6.28 | 0.54 |
| SPC5.3 | 24.50 | 33.28 | 7.37 | - | 8.15 | 1.28 | 0.05 | 5.98 | 0.58 |
| SPB8.3 | 24.71 | 33.51 | 6.96 | - | 8.14 | 0.27 | 0.01 | 3.92 | 0.49 |
| SPB6.3 | 24.88 | 33.56 | 7.23 | - | 8.16 | 0.31 | 0.07 | 5.69 | 0.77 |
| SPG4.3 | 24.92 | 33.62 | 6.67 | 0.14 | 8.18 | 0.76 | 0.07 | 4.89 | 0.60 |
| SPC7.3 | 25.01 | 33.68 | 6.86 | - | 8.15 | 0.32 | 0.03 | 3.91 | 0.63 |
| SUD7.3 | 31.23 | 31.61 | 4.29 | 0.09 | 8.12 | 0.35 | - | 0.16 | 0.25 |
| SUD5.3 | 31.28 | 30.99 | 6.82 | 0.06 | 8.19 | 0.55 | - | 0.18 | 0.23 |
| SUE6.3 | 31.43 | 30.44 | 7.12 | 0.09 | 8.12 | 0.70 | - | 0.16 | 0.23 |
| SUG4.3 | 31.43 | 32.58 | 6.65 | 0.02 | 8.12 | 0.29 | - | 0.19 | 0.27 |
| SUG2.3 | 31.45 | 32.52 | 6.86 | 0.05 | 8.12 | 2.08 | 0.24 | 0.23 | 1.24 |
| SUB2.3 | 31.58 | 28.79 | 6.64 | 0.07 | 8.04 | 2.32 | 0.01 | 0.15 | 0.39 |
| SUB4.3 | 31.58 | 30.95 | 6.25 | 0.09 | 8.19 | 0.60 | 0.01 | 0.13 | 0.44 |
| SUA1.3 | 31.62 | 29.82 | 6.55 | 0.10 | 8.20 | 0.96 | - | 0.20 | 0.23 |
| SUC3.3 | 31.70 | 30.83 | 7.01 | 0.15 | 8.25 | 0.03 | - | 0.17 | 0.41 |
| SUB6.3 | 31.77 | 32.05 | 6.37 | 0.03 | 8.22 | 0.51 | 0.02 | 0.11 | 0.44 |
| SUC5.3 | 31.81 | 31.65 | 6.56 | 0.45 | 8.21 | 0.15 | - | 0.11 | 0.34 |
| SUC1.3 | 31.84 | 27.72 | 6.29 | 0.23 | 8.12 | 0.88 | 0.06 | 0.14 | 0.65 |
| SUF1.3 | 31.94 | 32.04 | 7.10 | 0.06 | 8.18 | 1.84 | 0.03 | 0.18 | 0.27 |
| SUB8.3 | 32.07 | 31.60 | 6.63 | 0.11 | 8.20 | 0.14 | 0.03 | 0.13 | 0.85 |
| SUA4.3 | 32.11 | 29.41 | 6.59 | 0.07 | 8.19 | 0.18 | - | 0.13 | 0.47 |
| SUA6.3 | 32.23 | 31.60 | 6.26 | - | 8.18 | 0.18 | - | 0.11 | 0.34 |
| WIA1.3 | 22.43 | 29.82 | 15.38 | 0.38 | 8.31 | 2.27 | 0.02 | 0.08 | 0.12 |
| WIB2.3 | 23.24 | 31.68 | 15.44 | 0.26 | 8.20 | 1.00 | 0.02 | 0.14 | 0.14 |
| WIE6.3 | 23.64 | 30.85 | 15.40 | 0.30 | 8.13 | 1.15 | 0.02 | 0.28 | 0.16 |
| WIC1.3 | 23.81 | 31.35 | 15.30 | - | 8.19 | 2.24 | - | 0.11 | 0.17 |
| WID5.3 | 24.23 | 31.62 | 15.41 | 0.01 | 8.20 | 3.69 | 0.02 | 0.11 | 0.16 |
| WIF1.3 | 24.24 | 31.05 | 15.23 | 0.14 | 8.24 | 4.07 | 0.01 | 0.14 | 0.21 |
| WIC3.3 | 24.29 | 31.70 | 15.56 | 0.07 | 8.19 | 1.37 | - | 0.16 | 0.17 |
| WIB4.3 | 24.58 | 32.17 | 15.45 | 0.16 | 8.20 | 1.22 | 0.01 | 0.17 | 0.15 |
| WIB6.3 | 24.68 | 32.20 | 15.10 | 0.36 | 8.18 | 0.65 | - | 0.11 | 0.17 |
| WIA4.3 | 24.73 | 32.20 | 15.23 | 0.37 | 8.20 | 0.33 | 0.03 | 0.14 | 0.18 |
| WIC5.3 | 24.78 | 32.00 | 15.37 | 0.04 | 8.19 | 0.72 | 0.02 | 0.12 | 0.23 |
| WID7.3 | 24.92 | 31.49 | 15.42 | - | 8.23 | 0.87 | - | 0.14 | 0.17 |
| WIG4.3 | 25.13 | 31.49 | 15.33 | 0.09 | 8.21 | 0.53 | 0.01 | 0.14 | 0.17 |
| WIB8.3 | 25.44 | 32.22 | 15.11 | 0.26 | 8.22 | 0.45 | - | 0.09 | 0.17 |
| WIA6.3 | 25.53 | 32.34 | 15.44 | 0.27 | 8.18 | 0.34 | 0.02 | 0.10 | 0.13 |

SP: spring, SU: summer, WI: winter.
