# Supplementary material for: Warm‐Water Regimes Influence Microbial Diversity and Ecological Functions in Subtropical Gulf
Source: Ecol Evol. 2026 Apr 9;16(4):e73435. doi: 10.1002/ece3.73435 (PMC13063387; doi:10.1002/ece3.73435)
Supplement: Supplementary file 2 — Data S1: ece373435‐sup‐0002‐SupplementaryInformationCode.docx. [file ECE3-16-e73435-s002.docx]

**Supplementary Information (matlab and R code)**

**Warm-Water Regimes Influence Microbial Diversity and Ecological Functions in Subtropical Gulf**

**Qing He^1,a^, Laizhen Huang^1,a^, Chongqiu Huang^1^, Rajapakshalage Thashikala Nethmini^1^, Jannatul Ferdoush^1^, Shengyao Zhou^2^, Gonglingxia Jiang^1^, Qinghua Hou^1^, Xiaolei Li^1^, Qingxiang Chen^1^, Ke Dong^3^, Lingling Xie^1^, Nan Li^1*^**

^1^Key Laboratory of Climate, Resources and Environment in Continental Shelf Sea and Deep Sea of Department of Education of Guangdong Province, Department of Oceanography, Key Laboratory for Coastal Ocean Variation and Disaster Prediction, College of Ocean and Meteorology, Guangdong Ocean University, Zhanjiang 524088, China

^2^College of Environmental Science and Engineering, Guilin University of Technology, Guilin, China

^3^Department of Biological Sciences, Kyonggi University, 154-42, Gwanggyosan-ro, Yeongtong-gu, Suwon-si, Gyeonggi-do 16227, South Korea; Republic of Korea

**Water temperature distribution map(matlab code)**

data = readtable('23-wi-3.txt', 'Delimiter', '\t');

lon_station = double(data.lon);

lat_station = double(data.lat);

temp_station = double(data.Temp);

lon = ncread('F:\matlab-data\pingmian\ETOPO2v2g_f4.nc', 'x');

lat = ncread('F:\matlab-data\pingmian\ETOPO2v2g_f4.nc', 'y');

Bed = ncread('F:\matlab-data\pingmian\ETOPO2v2g_f4.nc', 'z');

lon(1:5401) = lon(1:5401) + 360;

K = Bed;

K(1:5401,:) = Bed(5401:end,:);

K(5402:end,:) = Bed(1:5400,:);

L = lon;

L(1:5400,:) = lon(5402:end,:);

L(5401:end,:) = lon(1:5401,:);

lon = L;

Bed = K;

A = find(lon == 108);

B = find(lon == 110);

C = find(lat == 20);

D = find(lat == 22);

Dep = Bed(A:B, C:D);

lon1 = lon(A:B);

lat1 = lat(C:D);

[xq, yq] = meshgrid(108:0.02:110, 20:0.02:22);

[lat1_mesh, lon1_mesh] = meshgrid(double(lat1), double(lon1));

dep_vq = griddata(lon1_mesh, lat1_mesh, Dep, xq, yq, 'natural');

dep_vq(dep_vq > 10000) = NaN;

F = scatteredInterpolant(lon_station, lat_station, temp_station, 'natural', 'none');

Temp_grid = F(xq, yq);

Temp_grid(Temp_grid < 20 | Temp_grid > 35) = NaN;

figure('Position', [100, 100, 800, 600]);

contourf(xq, yq, Temp_grid, 20, 'LineColor', 'none');

colormap(gca, jet);

caxis([22 25]);

colorbar;

hold on;

contour(xq, yq, dep_vq, [0 0], 'LineColor', 'k', 'LineWidth', 1.5);

scatter(lon_station, lat_station, 35, 'k', 'filled');

xlabel('Longitude (°E)');

ylabel('Latitude (°N)');

title('Sea Surface Temperature with Coastline (from ETOPO2)');

xlim([108 110]);

ylim([20 22]);

axis equal tight;

exportgraphics(gcf, 'sst_with_etopo_coastline.png', 'Resolution', 300);

disp('save as sst_with_etopo_coastline.png');

**Taxonomic composition ternary diagram**

library(Ternary)

dat2 <- dat

dat2$sum <- apply(dat1[2:4], 1, sum)

dat2$RS <- 100 * dat2$RS/dat2$sum

dat2$BS <- 100 * dat2$BS/dat2$sum

dat2$OR <- 100 * dat2$OR/dat2$sum

otu <- list()

for (i in 1:nrow(dat2)) {

otu[[i]] <- as.vector(unlist(dat2[i,c(2:4)]))

names(otu)[i] <- as.vector(dat2[i,1])

}

dat2[which(dat2$rich == 'RS'),'color'] <- 'red'

dat2[which(dat2$rich == 'BS'),'color'] <- 'blue'

dat2[which(dat2$rich == 'OR'),'color'] <- 'green3'

#dat2[which(dat2$rich == '0'),'color'] <- 'gray'

color <- c()

for (i in 1:nrow(dat2)) {

color[i] <- dat2[i,'color']

names(color)[i] <- as.vector(dat2[i,1])

}

size = c()

for (i in 1:nrow(dat2)) {

size[i] <- (apply(dat1[i,2:4], 1, mean))^0.4 / 5

names(size)[i] <- as.vector(dat2[i,1])

}

png('TernaryPlot.png', width = 2000, height = 2000, res = 300, units = 'px')

TernaryPlot(atip = 'RS', btip = 'BS', ctip = 'OR', col = NA, grid.col = 'gray', grid.minor.col = NA, axis.col = 'black')

AddToTernary(points, otu, col = color, cex = size, pch = 20)

dev.off()

**Alpha diversity analysis**

library(vegan)

goods <-

function(com){

no.seqs <- rowSums(com)

sing <- com==1

no.sing <- apply(sing, 1, sum)

goods <- 100*(1-no.sing/no.seqs)

goods.sum <- cbind(no.sing, no.seqs, goods)

goods.sum <- as.data.frame(goods.sum)

return(goods.sum)

}

table1<- read.table(file=file.choose(),sep=",",header=T,row.names=1)

otu=table1

otu=t(otu)

Shannon=diversity(otu,"shannon")

Simpson=diversity(otu,"simpson")

insimp=diversity(otu,"inv")

ss=specnumber(otu)

est=estimateR(otu)

est=t(est)

est1=est[,1:4]

coverage1=goods(otu)

Coverage=coverage1[,c("goods")]

alph=data.frame(Shannon,Simpson,est1,Coverage,check.names = T)

write.csv(alph, file="BBG16S_alph_acID.csv")

**NMDS**

library(vegan)

library(ggrepel)

library(ggplot2)

otu1<- read.table(file="2023_BBG_16S_HQ.otu.csv",sep=",",header=T,row.names=1)

group=read.table(file="2023_BBG_16S_HQ.newgroup.csv",sep=",",header=T,row.names=1)

otu=otu1[match(row.names(group),row.names(otu1)),]

otu <- otu1[match(rownames(group), rownames(otu1)), ]

otu <- apply(otu, 2, as.numeric)

rownames(otu) <- rownames(group)

otu <- otu[rowSums(otu) > 0, ]

if(any(is.na(otu))) {

otu[is.na(otu)] <- 0

}

otu.nmds <- metaMDS(otu, distance = 'bray', k = 4)

NMDS = data.frame(MDS1 = otu.nmds$points[,1], MDS2 = otu.nmds$points[,2])

NMDS1=NMDS[match(row.names(group),row.names(NMDS)),]

ggtable3=data.frame(NMDS1,group,check.rows = T)

df3.1<-ggtable3[ggtable3$group=="SPL",][chull(ggtable3[ggtable3$group=="SPL",c("MDS1","MDS2")]),]

df3.2<-ggtable3[ggtable3$group=="SUL",][chull(ggtable3[ggtable3$group=="SUL",c("MDS1","MDS2")]),]

df3.3<-ggtable3[ggtable3$group=="WIL",][chull(ggtable3[ggtable3$group=="WIL",c("MDS1","MDS2")]),]

df3.4<-ggtable3[ggtable3$group=="SPM",][chull(ggtable3[ggtable3$group=="SPM",c("MDS1","MDS2")]),]

df3.5<-ggtable3[ggtable3$group=="SUM",][chull(ggtable3[ggtable3$group=="SUM",c("MDS1","MDS2")]),]

df3.6<-ggtable3[ggtable3$group=="WIM",][chull(ggtable3[ggtable3$group=="WIM",c("MDS1","MDS2")]),]

df3.7<-ggtable3[ggtable3$group=="SPH",][chull(ggtable3[ggtable3$group=="SPH",c("MDS1","MDS2")]),]

df3.8<-ggtable3[ggtable3$group=="SUH",][chull(ggtable3[ggtable3$group=="SUM",c("MDS1","MDS2")]),]

df3.9<-ggtable3[ggtable3$group=="WIH",][chull(ggtable3[ggtable3$group=="WIH",c("MDS1","MDS2")]),]

ggpc3 <- ggplot(ggtable3,aes(MDS1,MDS2,colour=group))

ggpc3

p3<-ggpc3 + geom_point(data = ggtable3,aes(MDS1,MDS2,shape=ggtable3$group,fill=ggtable3$group),size=1.5) +

scale_shape_manual(values = c(16,16,16,16,16,16,16,16,16))+

ggtitle("NMDS",paste('Stress =',round(otu.nmds$stress, 4))) +

geom_hline(yintercept =0,colour='grey80', linetype='dashed') +

geom_vline(xintercept =0,colour='grey80', linetype='dashed') +

#geom_text_repel(data = ggtable3,aes(MDS1,MDS2,label=row.names(ggtable3)),size=4)+

theme_bw()+theme(panel.grid=element_blank())+

geom_polygon(data=df3.1,aes(x=MDS1,y=MDS2,group=group),

color="#61AC85",fill="#61AC85",alpha=0.2,size=0.1)+

geom_polygon(data=df3.2,aes(x=MDS1,y=MDS2,group=group),

color="#61AC85",fill="#61AC85",alpha=0.2,size=0.1)+

geom_polygon(data=df3.3,aes(x=MDS1,y=MDS2,group=group),

color="#61AC85",fill="#61AC85",alpha=0.2,size=0.1)+

geom_polygon(data=df3.4,aes(x=MDS1,y=MDS2,group=group),

color="#EA8958",fill="#EA8958",alpha=0.2,size=0.1)+

geom_polygon(data=df3.5,aes(x=MDS1,y=MDS2,group=group),

color="#EA8958",fill="#EA8958",alpha=0.2,size=0.1)+

geom_polygon(data=df3.6,aes(x=MDS1,y=MDS2,group=group),

color="#EA8958",fill="#EA8958",alpha=0.2,size=0.1)+

geom_polygon(data=df3.7,aes(x=MDS1,y=MDS2,group=group),

color="#ED2F2A",fill="#ED2F2A",alpha=0.2,size=0.1)+

geom_polygon(data=df3.8,aes(x=MDS1,y=MDS2,group=group),

color="#ED2F2A",fill="#ED2F2A",alpha=0.2,size=0.1)+

geom_polygon(data=df3.9,aes(x=MDS1,y=MDS2,group=group),

color="#ED2F2A",fill="#ED2F2A",alpha=0.2,size=0.1)+

scale_x_continuous(limits = c(-0.8,0.75),breaks = c(-0.75,-0.5,-0.25,0,0.25,0.5,0.75))+

scale_y_continuous(limits = c(-0.75,0.75),breaks = c(-0.75,-0.5,-0.25,0,0.25,0.5,0.75))+

theme_bw()+theme(panel.grid=element_blank())

p3

ggsave('NMDS_nifH-2023_BBG_16S_HQ.pdf',p3, width =7, height =5,limitsize = FALSE)

df8<-ggtable3[ggtable3$group=="SPL",][chull(ggtable3[ggtable3$group=="SPL",c("MDS1","MDS2")]),]

df9<-ggtable3[ggtable3$group=="SUL",][chull(ggtable3[ggtable3$group=="SUL",c("MDS1","MDS2")]),]

df10<-ggtable3[ggtable3$group=="WIL",][chull(ggtable3[ggtable3$group=="WIL",c("MDS1","MDS2")]),]

df11<-ggtable3[ggtable3$group=="SPM",][chull(ggtable3[ggtable3$group=="SPM",c("MDS1","MDS2")]),]

df12<-ggtable3[ggtable3$group=="SUM",][chull(ggtable3[ggtable3$group=="SUM",c("MDS1","MDS2")]),]

df13<-ggtable3[ggtable3$group=="WIM",][chull(ggtable3[ggtable3$group=="WIM",c("MDS1","MDS2")]),]

df14<-ggtable3[ggtable3$group=="SPH",][chull(ggtable3[ggtable3$group=="SPH",c("MDS1","MDS2")]),]

df15<-ggtable3[ggtable3$group=="SUH",][chull(ggtable3[ggtable3$group=="SUH",c("MDS1","MDS2")]),]

df16<-ggtable3[ggtable3$group=="WIH",][chull(ggtable3[ggtable3$group=="WIH",c("MDS1","MDS2")]),]

ggpc3 <- ggplot(ggtable3,aes(MDS1,MDS2,colour=group))

ggpc3

p33<-ggpc3 + geom_point(data = ggtable3,aes(MDS1,MDS2,shape=ggtable3$group,fill=ggtable3$group),size=1.5) +

scale_shape_manual(values = c(16,16,16,16,16,16,16,16,16))+

ggtitle("NMDS",paste('Stress =',round(otu.nmds$stress, 4))) +

geom_hline(yintercept =0,colour='grey80', linetype='dashed') +

geom_vline(xintercept =0,colour='grey80', linetype='dashed') +

geom_text_repel(data = ggtable3,aes(MDS1,MDS2,label=row.names(ggtable3)),size=4)+

theme_bw()+theme(panel.grid=element_blank())+

geom_point(data=ggtable3,aes(x=MDS1,y=MDS2,color=group,shape=group),size=3)+

theme_bw()+

theme(panel.background = element_blank(),

panel.grid = element_blank(),

legend.title = element_text(hjust=0.5))+

scale_color_manual(values = c("#008080","#ffa500","#8b008b","#7cae00","#b79f00","#FED71A","#00bfc4","#de8c00","#a7a8bd"))+

geom_polygon(data=df8,aes(x=MDS1,y=MDS2,group=group),

color="#008080",fill="#008080",alpha=0.2,size=1)+

geom_polygon(data=df9,aes(x=MDS1,y=MDS2,group=group),

color="#ffa500",fill="#ffa500",alpha=0.2,size=1)+

geom_polygon(data=df10,aes(x=MDS1,y=MDS2,group=group),

color="#8b008b",fill="#8b008b",alpha=0.2,size=1)+

geom_polygon(data=df10,aes(x=MDS1,y=MDS2,group=group),

color="#7cae00",fill="#7cae00",alpha=0.2,size=1)+

geom_polygon(data=df10,aes(x=MDS1,y=MDS2,group=group),

color="#b79f00",fill="#b79f00",alpha=0.2,size=1)+

geom_polygon(data=df10,aes(x=MDS1,y=MDS2,group=group),

color="#FED71A",fill="#FED71A",alpha=0.2,size=1)+

geom_polygon(data=df10,aes(x=MDS1,y=MDS2,group=group),

color="#00bfc4",fill="#00bfc4",alpha=0.2,size=1)+

geom_polygon(data=df10,aes(x=MDS1,y=MDS2,group=group),

color="#de8c00",fill="#de8c00",alpha=0.2,size=1)+

geom_polygon(data=df10,aes(x=MDS1,y=MDS2,group=group),

color="#a7a8bd",fill="#a7a8bd",alpha=0.2,size=1)+

theme_bw()+theme(panel.grid=element_blank())

p33

ggsave('NMDS_nifH-3loc-3group-20250602-zy.pdf',p33, width =15, height =12,limitsize = FALSE)

**Heatmap**

library(psych)

library(ggplot2)

library(pheatmap)

x<- read.table(file=file.choose(),sep=",",header=T,row.names=1)

y <- psych::corr.test(x,method = "spearman")

r<-y$r

p<-y$p

sig_label<-function(jp){ifelse(jp<0.001,"***",ifelse(jp<0.01,"**",ifelse(jp<0.05,"*","")))}

sig_matrix<-sig_label(p)

r=round(r,2)

r<-r[-c(1:15),-c(16:29)]

sig_matrix<-sig_matrix[-c(1:15),-c(16:29)]

bk <- c(seq(-1,-0.1,by=0.1),seq(0,1,by=0.1))

p<- pheatmap(r,fontsize=10,border_color = "black",

color = c(colorRampPalette(colors = c("blue","white"))(length(bk)/2),colorRampPalette(colors = c("white","red"))(length(bk)/2)),

display_numbers = sig_matrix,fontsize_row =10,fontsize_col = 10,

fontsize_number = 20,

legend_breaks=seq(-1,1,0.5),

breaks=bk,

cluster_rows=F,cluster_cols=F)

p

ggsave('BBG16S-heatmap.pdf',p,width=10,height=8,limitsize=FALSE)

**Random forest**

library(randomForest)

#install.packages("randomForest")

g<- read.table(file="phytoplankton bbg18s-20250602.csv",sep=",",header=T,row.names=1)

g$group <- factor (g$group)

set.seed(315)

g.rf = randomForest(group ~ ., data=g, importance=TRUE, proximity=TRUE)

print(g.rf)

round(importance(g.rf), 2)

varImpPlot(g.rf)

result <- round(importance(g.rf), 2)

write.csv(result, file="s_result-gini.csv")

taxa_table<- subset(g, select = -c(group))

ginirank<- read.table(file="ginirank.csv",sep=",",header=T,row.names=1)

taxa_table<-t(taxa_table)

taxa_table1=taxa_table[match(row.names(ginirank),row.names(taxa_table)),]

taxa_table2=head(taxa_table1,12)

write.csv(taxa_table2, file="taxa_gini.csv")

taxa_gini_top20<-read.table(file="taxa_gini.csv",header=T,sep=",")

library(reshape2)

taxa_gini_top20.melt<- melt(taxa_gini_top20)

write.csv(taxa_gini_top20.melt, file="taxa_gini.melt.csv")

g_table<-read.table(file="taxa_gini.melt.csv",header=T,sep=",")

library(ggplot2)

p1<-ggplot(data=g_table, aes(x=taxa,y=value))+

geom_boxplot(aes(fill=group),outlier.shape = NA)+

theme_bw()+theme(panel.grid=element_blank())+

theme_bw()+

coord_flip()

p1

ggsave('xiangxian.pdf',

p1,width=8,height=7,limitsize=FALSE)
